# Does CSR performance improve corporate immunity to the COVID-19 pandemic? Evidence from China's stock market

**DOI:** 10.3389/fpubh.2022.956521

**Published:** 2022-08-16

**Authors:** Jing Tian, Xiuxiu Wang, Yanqiu Wei

**Affiliations:** School of Economics, Tianjin University of Commerce, Tianjin, China

**Keywords:** CSR, corporate immunity, stock return, COVID-19 pandemic, PSM-DID

## Abstract

This paper studies the role of corporate social responsibility (CSR) performance on corporate financial performance during the COVID-19 by examining a sample of Chinese listed firms. Based on the PSM-DID methodology, we find that the pandemic-induced decline in stock returns is stronger with more CSR engagement. The results remain robust even after the dynamic effect test and placebo test. It means CSR performance does not improve Chinese corporate immunity to the pandemic. This inadequate response of CSR could be due to the “relatively few good things effect”. Furthermore, our study indicates that increasing awareness of responsible investment and improving the quality of CSR disclosure could facilitate CSR engagement in China.

## Introduction

The global economic crisis precipitated by COVID-19 is unlike any other in history. It resulted from a public health emergency that severely hampered global economic activity. While countries worldwide have responded quickly to the stagnation and decline in development brought about by the COVID-19 shock, global economies have unavoidably suffered from the economic downturn and massive stock market fluctuations (1). Simultaneously, stock return volatility varies significantly across countries and firms, even within the same country and industry. These observations prompt discussion of the heterogeneity of responses to COVID-19 based on the country and firm characteristics.

Which characteristics endow some firms with more excellent resistance to the pandemic than others? Recent studies have explored corporate immunity in a variety of ways. According to Zaremba et al. (2), stock markets in countries with low unemployment rates, lower valuations relative to expected profits, and conservative investment policies are more immune to the pandemic; additionally, firm government policies can provide support for the stock market. Pagano et al. (3) demonstrate that during the COVID-19 crisis, companies less impacted by social distancing earned a higher rate of return. Ding et al. (4) find that firms with healthy financial conditions, less affected by the international supply chain, less entrenched executives, and more CSR activities performed better during the pandemic. In terms of corporate performance, the impact of CSR is far from conclusive. Renneboog et al. (5) note that existing research does not declare unequivocally that investors are prepared to accept sub-optimal financial performance to aspiring to social or moral objectives. Some findings believe that executives participate in CSR activities to enhance their personal reputation and credibility at the expense of other stakeholders (6, 7). Employing a sample of 25 international airlines observed from 2010 to 2016, Lahouel et al. (8) document that CSR has a significant negative impact on corporate performance. However, Lins, Servaes, and Tamayo (9) conduct a survey on US companies throughout the global financial crisis and show that markets respond favorably to CSR. Albuquerque et al. (10) develop a theoretical framework to clarify that CSR activities can increase product differentiation and customer loyalty, thereby reducing companies' sensitivity to economic recession.

CIVID-19 forced people to rethink their development model. Nowadays, many countries prioritize sustainable and green issues, and CSR is being widely embraced. However, empirical research on the role of CSR during the COVID-19 crisis remains limited, especially within a single country or region. To continue implementing CSR activities, more evidence is needed to evaluate the effectiveness of those activities. This study aims to extend the existing literature by examining how CSR affected Chinese listed firms' performance during the COVID-19 pandemic. Our knowledge indicates that this effect has not yet been investigated in the literature.

## CSR activities in China's capital market

As a critical component of Chinese enterprises, listed firms have a significant impact on the long-term development of capital markets and society. The China Securities Regulatory Commission has conducted extensive exploration into developing a social responsibility investment system to incentivize the CSR behavior of listed firms. Since 2006, Shenzhen and Shanghai stock Exchanges have required listed firms to fulfill their social responsibilities actively and voluntarily disclose corporate social responsibility reports. In 2009, Shanghai and Shenzhen Stock Exchanges launched responsibility indexes to track the performance of listed firms' social responsibilities. Each year, the top 100 firms with the highest value of social contribution per share are selected as constituent stocks respectively.^1^ Following CSR regulations and guidelines, lots of listed companies disclose, explain, and emphasize their social responsibility strategies. However, the proportion of listed firms that disclose CSR reports was only 26% of the total number of listed firms at the end of 2020. Most CSR reports are based on non-monetary and qualitative data that do not adequately address the actual needs of stakeholders. In this case, China's CSR research is constrained by two factors. First, the average quality of CSR activities is relatively low, hindering Chinese capital markets from responding to CSR effectively. Second, it is challenging to quantify the fulfillment of CSR comprehensively. Currently, CSR scores of listed firms are released by a couple of third-party agencies. Their evaluations are based on CSR reports of listed firms, which may not objectively reflect listed firms' actual CSR activities. In addition, concerning some qualitative indicators of CSR, there appear to be differences in the views of these assessment organizations, leading them to assign different CSR scores to the same company.

## Data and identification

### Data description

Based on the above analysis, we prefer 146 constituent firms from Shanghai and Shenzhen Stock Exchange's social responsibility indexes as the treated group, excluding newly selected firms within the last 3 years. This is done to establish a relationship between CSR and enterprise performance. This treatment group selection has the following two advantages: (i) Shanghai and Shenzhen Stock Exchanges' social responsibility indexes are arguably the most respected and prominent available measure for CSR performance, which eliminates the possibility of CSR score distortion; (ii) social responsibility information about constituent firms is fully disclosed, resulting in a low degree of information asymmetry between corporations and their stakeholders, making it easy to identify and isolate the causal effect of CSR. Although the sample companies are only a small part of all listed companies, the causal relationship between CSR and corporate market performance should be shown through these sample companies.

The dividend-adjusted monthly stock return (in percentage) is used as the dependent variable to assess listed companies' market performance, while the dividend-adjusted monthly abnormal stock return is used to test robustness. The abnormal return is equal to the monthly stock returns of each firm minus the beta multiplied by the monthly return of the domestic markets (valued-weighted), with beta estimated using yield data from the previous 250 trading days. As suggested by Ding et al. (4), we use six fundamental financial characteristics from prior financial statements as control variables. Volatility is equal to the logarithmic rate of return on the stock over the previous 250 trading days. Total liabilities divided by total assets equals Lev. Cash flow is calculated as the ratio of operating cash flow to total assets. ROA is the ratio of net assets to total assets. Size is equal to the natural logarithm of the total assets' book value. BM is abbreviated as the ratio of book value to market value. We extract data about stock returns and corporate financials from the China Stock Market and Accounting Research Database (CSMAR).

### Empirical strategy

**Figure 1:**
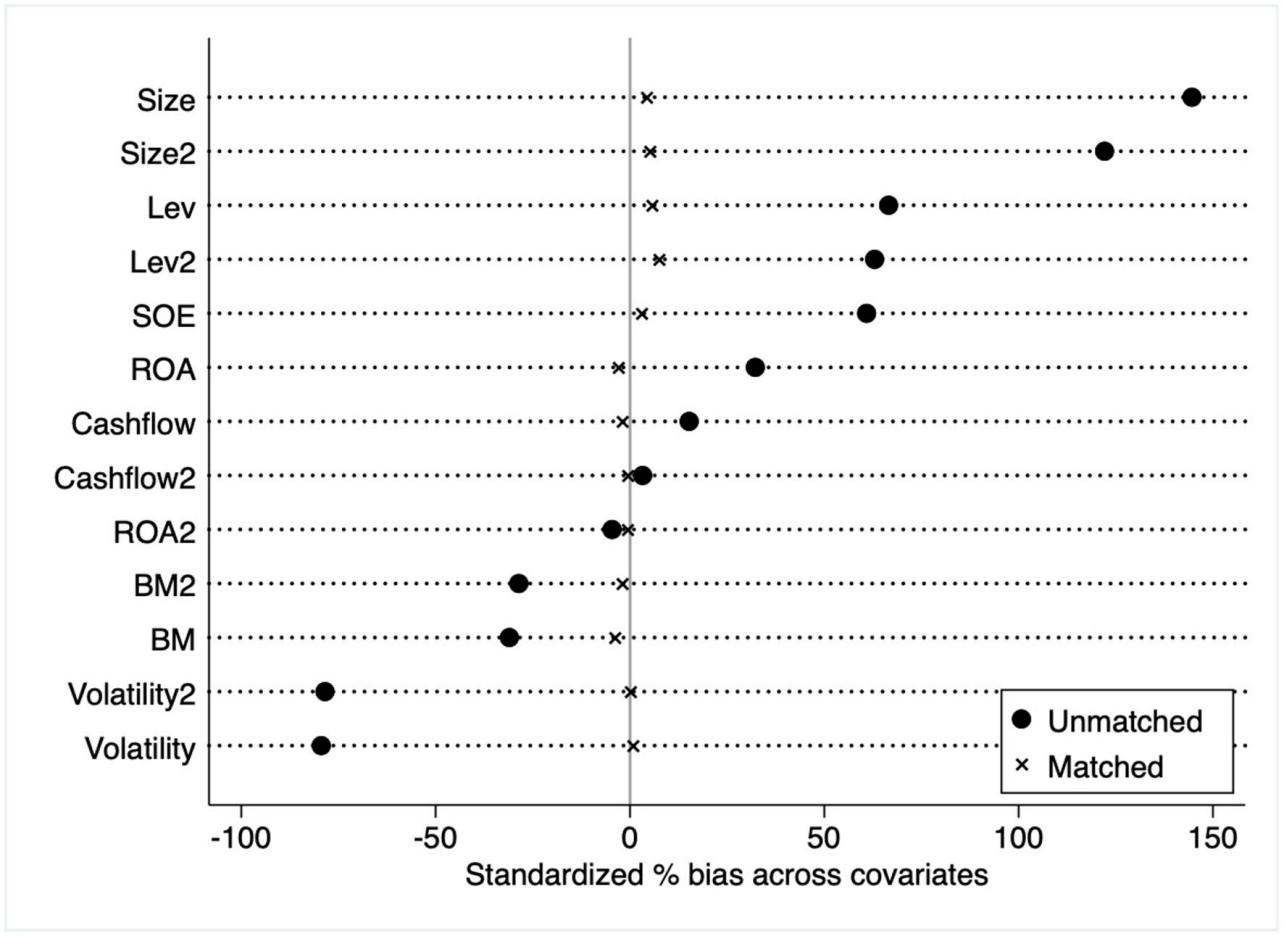
Covariates balance test, unmatched vs. matched. The Y-axis in the figure is the covariates entering the propensity index model, including the control variables (e.g., Volatility, Lev, ROA, Cashflow, Size, and BM) and their square terms (e.g., Volatility2, Lev2, ROA2, Cashflow2, Size2, and BM2) and the type of ownership (SOE). The X-axis is the standardized bias across covariates.

Drawing on the study method of emergency in Abadie and Dermisi (11), We treat the lockdown of Wuhan on January 23, 2020, as an exogenous shock (this is not only the final trading day of January but also the final trading day before the Chinese Lunar New Year) and use a difference-in-difference model to identify the causal relationship between CSR and stock returns. By effectively separating the dual impact of crisis and CSR on stock returns, it is possible to estimate more precise causal effects. To avoid selection bias and endogenous problems, we select the control group using propensity score matching (PSM). The treated group is similar to the control group in terms of all control variables, including Volatility, Leverage, Cash flow, ROA, Size, and BM. Given that market response to CSR vary according to firm ownership type (12), we also consider firm ownership type, state-owned enterprises (SOE), and non-SOE measures. Figure 1 depicts the result of the covariates balance test. We could see that the two groups of listed firms are extremely well matched across all covariates.

We estimate difference-in-difference regression to compare the monthly stock returns of treated and control groups from January 2019 to December 2020:


(1) $$\begin{array}{r} y_{it}=\alpha_{0}+\alpha_{i}^{static}D_{it}+\alpha^{{}^{\prime }}controls+u_{i}+\lambda_{t}+\varepsilon_{it} \end{array}$$

The dependent variable, *y*_*it*_ is the monthly return or abnormal return. Where treatment dummy *D*_*it*_ is the core explanatory variable we care about. It is equal to one if the firm belongs to the control group and the time is after the outbreak of the epidemic, and it is zero otherwise. $a_{i}^{static}$is the average treatment effect. The matrix of control variables includes Volatility, Lev, ROA, Cashflow, Size, and BM. To determine whether the dependent variables satisfy the prior parallel trend hypothesis and investigate the time distribution of treatment effects, we estimate the dynamic effect of CSR using the event study method proposed by Jacobson and Sullivan (13):


(2) $$\begin{array}{r} y_{it}=\alpha_{0}+\sum {\;}_{l}\alpha_{l}D_{tl}+\alpha^{{}^{\prime }}controls+u_{i}+\lambda_{t}+\varepsilon_{it} \end{array}$$

where, *D*_*tl*_ denotes a set of dummies, and *l* denotes the month relative to the outbreak date (e.g., *D*_*t*1_ denotes whether it is the month following the event, whereas *D*_*t*0_ denotes whether it is the month when COVID-19 outbroke). If there are no significant treatment effects before COVID-19, the parallel trend hypothesis is valid. Each month following COVID-19, the coefficients can be used to describe the dynamic changes in the CSR treatment effects over time.

## Results

### Results of main regression

The main regression is summarized in Table 1. Monthly returns are used as dependent variables in the first two columns of Table 1. While column 1 controls only firm and time fixed effects, column 2 includes additional control variables; the average treatment effect remains statistically significant at 1%. The empirical evidence demonstrates that CSR significantly negatively affects stock returns during the COVID-19 pandemic. In terms of magnitude, the average monthly returns of corporates with high levels of CSR are 0.8 percent lower than that of their peers. Columns 3 and 4 demonstrate that the average treatment effects remain statistically and economically significant by utilizing the monthly abnormal return rate. Thus, our findings support Friedman's trade-off theory (14). According to the trade-off theory, corporations that engage in CSR efforts incur opportunity costs that adversely affect profitability, competitiveness, and innovation capability. During the COVID-19 epidemic, investors place a premium on the intrinsic value of stocks or seek short-term gains from sentiment-driven stock price and thus react negatively to CSR practices. The findings imply that CSR performance does not improve corporate immunity to the pandemic.

**Table 1:** The effect of CSR on stock return: main regression.

	**(1)**	**(2)**	**(3)**	**(4)**
	**Monthly return**	**Monthly return**	**Abnormal return**	**Abnormal return**
D^static^	−0.016*** (0.003)	−0.008*** (0.003)	−0.017*** (0.003)	−0.007** (0.003)
Volatility		−0.025* (0.014)		−0.059*** (0.014)
Lev		−0.086** (0.031)		−0.193*** (0.032)
ROA		0.247** (0.041)		0.271*** (0.014)
Cashflow		−0.102 (0.114)		−0.079 (0.109)
Size		−0.055*** (0.011)		−0.070*** (0.012)
BM		−0.534*** (0.019)		−0.637*** (0.019)
Constant	0.029*** (0.001)	0.501*** (0.042)	0.001 (0.001)	0.636*** (0.043)
N	5,354	5,354	5,354	5,354
adj. R-sq	0.32	0.34	0.11	0.13

The table shows the impact of corporate social responsibility on stock returns. Monthly stock returns and monthly abnormal stock returns are the dependent variables. Both firm-fixed and time-fixed effects are controlled. Standard errors for firm-clustered robustness are given in parentheses. ^***^,^**^, and ^*^ denote the 1, 5, and 10% significance levels, respectively.

### Dynamic effects

**Figure 2:**
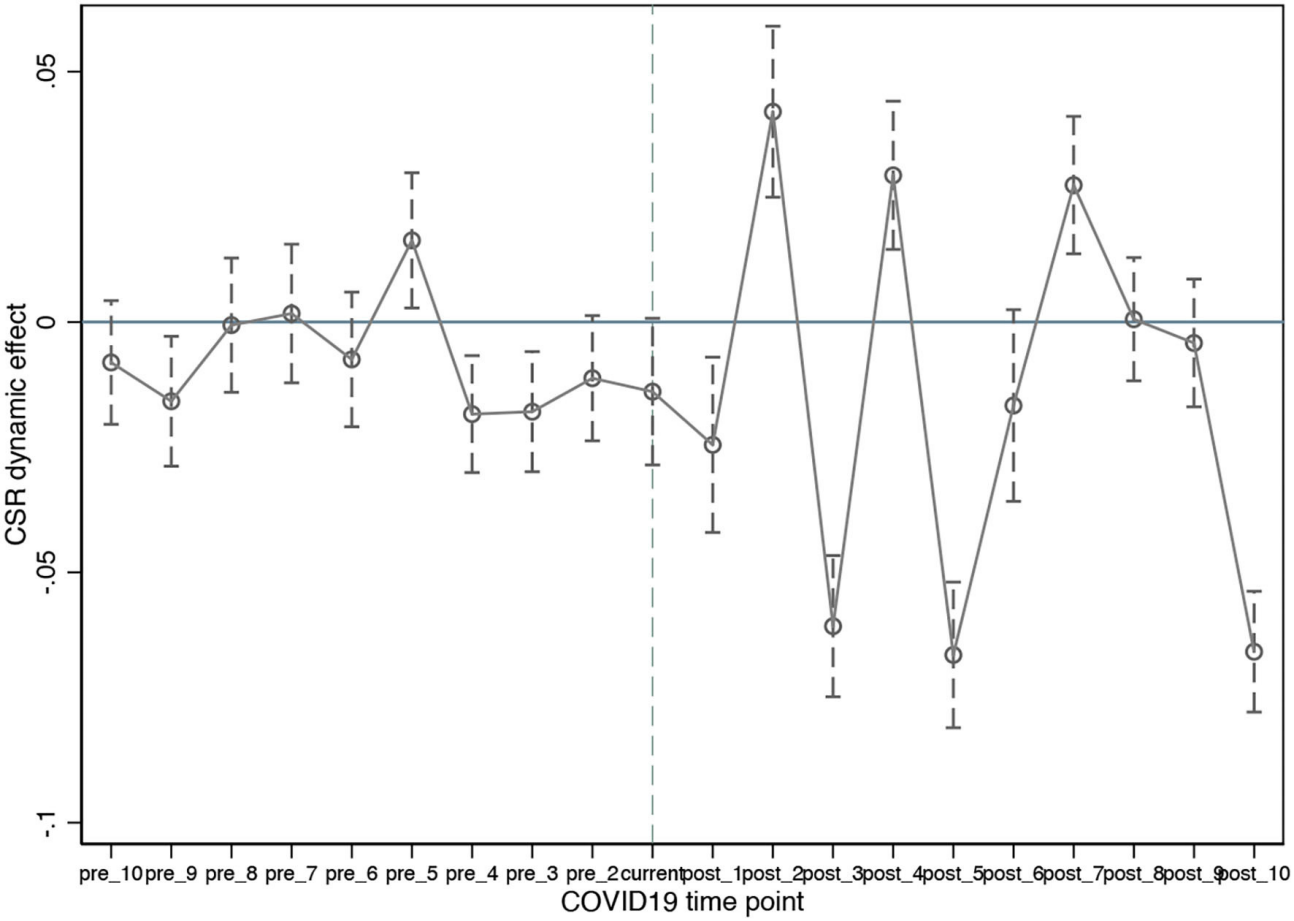
The dynamic effect of CSR on stock return. The figure is based on period −1, and the coefficients of other periods are relative values to that period. The solid points are the point estimates of the coefficients of each period, and the short vertical lines are the confidence intervals calculated using the robust standard errors of the individual-level clustering at the 95% significance level.

This section examines the evolution of CSR's effect prior to and following the COVID-19 pandemic. We examine dynamic effects by employing Eq (2). We analyze the period up to 10 months prior to and 10 months following the event, with a month before the event serving as the base period. The dynamic effects of CSR are depicted in Figure 2, along with their confidence intervals. As seen in the graph, most of the coefficients before the event are small and insignificant, indicating that the treated and control groups have comparable pre-treatment trends in their stock returns. By contrast, their stock returns diverge significantly following the event; the divergences persist for up to 10 months afterward. The findings confirm that China's stock market is incapable of positively pricing CSR activities.

### Placebo test

**Figure 3:**
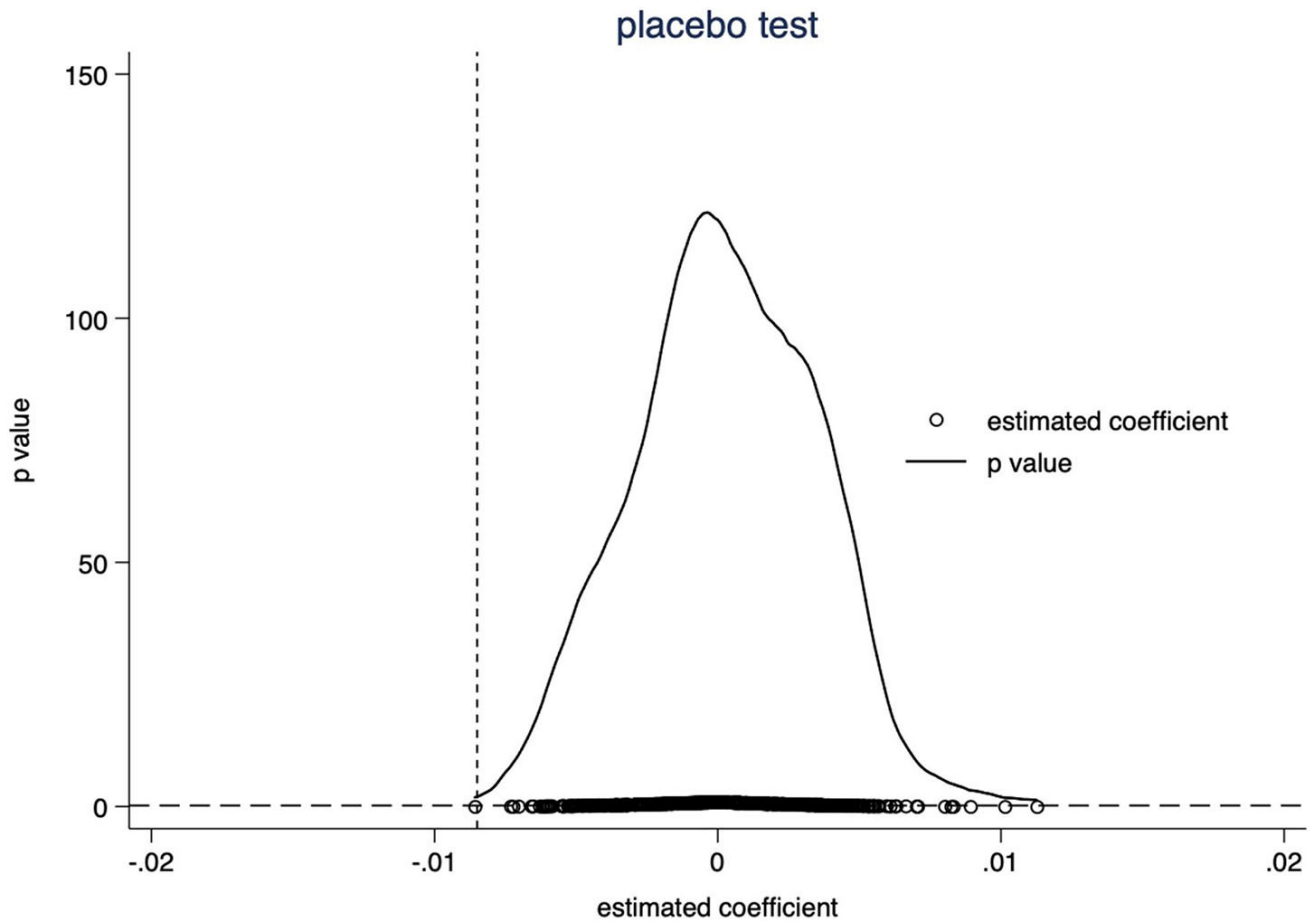
Placebo test. This figure depicts the distribution of estimated coefficients and corresponding *p*-values for 500 pseudo-treated samples. The X-axis depicts the estimated pseudo-treatment coefficients. The Y-axis depicts the *p*-values. The vertical dashed line depicts the natural treated effect of −0.008. The horizontal dashed line depicts the 10% significant level. As illustrated in the figure, the estimated coefficients are predominantly near zero, and the majority of estimated values have *p*-values greater than 0.1 (not significant at the 10 percent level).

To ensure that our methodology accurately captures stock returns solely driven by CSR and not by some omitted variables, we conduct a placebo test. We replace treated firms with pseudo-treated firms drawn at random from the entire sample of firms in the month of the pandemic's start. The regression coefficient is estimated repeatedly 500 times. The placebo plot in Figure 3 demonstrates that the average value of the estimated coefficients for 500 regressions is close to 0. In contrast, the nature coefficients in the basic regression, denoted by the dotted line on the left, are statistically significant. The placebo test verifies that the results of the main regression are not due to unobserved accidental factors.

## Discussion

Our findings do not support the claim that CSR increases corporate immunity. By contrast, Ding et al. (4) demonstrate that CSR has a positive effect on stock prices during the COVID-19 by examining 6,700 listed firms in 61 countries. Our study is different from theirs in three ways. First, in terms of sample country, we examine listed firms from China. Second, in terms of methodology, we conduct PSM-DID to ascertain the causal relationship between CSR and the stock market performance, using constituent firms of Responsibility Index as treated firms and their matched non-index-constituent ones as control firms. The advantage of this approach is that we can control for confounding factors that might affect the outcome and avoid CSR scores distortion, whereas Ding et al. (4) apply the fixed effect regression drawing on SCR scores. Third, in terms of timeline, we focus on the medium-term effects of CSR during 1 year of the pandemic using monthly stock returns, whereas Ding et al. (4) concentrate on short-term effects during the weeks from January 3 through May 22, 2020.

Concerning CSR activities such as ensuring worker safety, providing safe products, honoring informal agreements with suppliers, and environmental protection, it means that the corporation has committed to honoring its informal commitments. These activities can help strengthen the bond between the firm and its stakeholders. These strengthened relationships, in turn, aid in the retention of highly loyal employees and customers during the recession (4, 10, 15). From this vantage point, the stock prices of companies with a strong commitment to CSR should be more resilient to the epidemic. Nonetheless, several factors, including the conflict of interests between investors and executives and the credibility of CSR disclosures, continue to influence the CSR-corporate performance nexus (16, 17). For example, Ding et al. (4) also prove that CSR activities strengthen corporate immunity in societies that value them highly and in economies where social norms place a premium on human rights and the environment. CSR is more likely to increase loyalty and strengthen relationships with stakeholders in these economies.

Brammer and Millington (18) shed light on a U-shaped linkage between CSR and corporate financial performance, with higher financial performance being associated with extremely high or extremely low CSR. Corporates pursuing low-cost strategies (low CSR) or differentiated strategies (high CSR) are likely to outperform those stuck in the middle. But firms with poor social performers do best in the short run, while firms with good social performers do best over longer time horizons. From this point of view, the reason for the inadequate response to CSR, which puzzles China's capital market, could be due to the “relatively few good things effect.” Although CSR initially has a detrimental effect on corporate performance, this effect will be reversed once a certain level of CSR participation is reached, ultimately promoting profitability improvement.

Our findings not only add new research perspectives and empirical evidence to the pertinent literature but also help guide post-crisis CSR activities. Given that CSR is a crucial driver of sustainable development, the significance of our findings extends beyond its impact on corporate performance. In summary, our study implies that increasing awareness of responsible investment and improving the quality of CSR disclosure could lead to greater CSR engagement in China.

## Data availability statement

The original contributions presented in the study are included in the article/supplementary material, further inquiries can be directed to the corresponding author/s.

## Author contributions

JT: conceptualization, methodology, writing, and supervision. XW: investigation and data collection. YW: investigation and data curation. All authors contributed to the article and approved the submitted version.

## Funding

We acknowledge the funding from the Philosophy & Social Science Fund of Tianjin City, China. Award #: TJYY17-018 (Research on the Fund Guarantee for the Development of Home-based Elderly Care Services in Tianjin City).

## Conflict of interest

The authors declare that the research was conducted in the absence of any commercial or financial relationships that could be construed as a potential conflict of interest.

## Publisher's note

All claims expressed in this article are solely those of the authors and do not necessarily represent those of their affiliated organizations, or those of the publisher, the editors and the reviewers. Any product that may be evaluated in this article, or claim that may be made by its manufacturer, is not guaranteed or endorsed by the publisher.

## Footnotes

^1^The value of social contribution per share is calculated by adding the value created for other stakeholders such as taxes, salaries, interest payments, and donations to the earnings per share and deducting the cost of environmental pollution.
